# Knowledge and attitudes to vitamin D and sun exposure in elite New Zealand athletes: a cross-sectional study

**DOI:** 10.1186/s12970-014-0047-6

**Published:** 2014-09-17

**Authors:** Nicole Walker, Thomas D Love, Dane Francis Baker, Phillip Brian Healey, Jillian Haszard, Antony S Edwards, Katherine Elizabeth Black

**Affiliations:** Department of Human Nutrition, University of Otago, PO Box 56, Dunedin, 9054 New Zealand; College of Engineering, Swansea University, Swansea, UK; Chiefs Super Rugby Franchise, Hamilton, New Zealand; High Performance Sport New Zealand, Auckland, New Zealand

**Keywords:** Sun exposure, Cancer, Vitamin D, Athletes

## Abstract

**Background:**

Sun safety and vitamin D status are important for prolonged health. They are of particular interest to those working with athletes for whom for whom safe sun practices maybe limited.

The aim of this cross-sectional study was to describe the attitudes of elite New Zealand athletes to both vitamin D and sun exposure.

**Methods:**

110 elite New Zealand outdoor athletes volunteered to participate in an interview with a trained interviewer. The interviewer asked the athletes questions on their Vitamin D knowledge, attitudes and practices regarding sun exposure as well as their concerns about skin cancer.

**Results:**

Athletes were more concerned about their risk of skin cancer (66%) than their vitamin D status (6%). Although the majority (97%) were aware of Vitamin D and could identify the sun as a source (76%) only 17% could name another source of Vitamin D.

Only 10 (9%) reported always applying sunscreen before going out in the sun. No athlete reported reapplying sunscreen every hour and 25 suggesting that they never reapply sunscreen.

**Conclusions:**

Athletes are concerned about skin cancer however, their use of sunscreen is not optimal suggesting reapplication of sunscreen could be targeted in order to reduce the risk of sun cancer. Awareness of sources of Vitamin D other than the sun may also need to be improved potentially through educational interventions and possibly in conjunction with sun smart messages.

## Background

The Vitamin D status of athletes has been described for various athletic populations [1]. History shows that Vitamin D deficiency or insufficiency may be detrimental to performance [2-4] and more recent evidence suggests health implications for those with inadequate compared to those with an adequate serum vitamin D concentration [5]. Insufficient serum Vitamin D concentration has been associated with lower muscle strength and endurance capacity, further it is known that Vitamin D plays an important role in bone health [1,2,6]. However, the awareness of athletes to Vitamin D and their knowledge of its potential implications for health and performance is currently unknown. An understanding of an athlete’s awareness, knowledge and attitudes towards Vitamin D could help guide interventions aimed at ensuring adequate Vitamin D status in elite athletes.

Vitamin D is produced in the skin when ultraviolet B (UVB) radiation from sunlight converts cutaneous 7-dehydrocholesterol to previtamin D [7]. Given the importance of sun exposure for vitamin D synthesis and skin cancer risk, an athletes attitudes towards skin cancer risk may influence their vitamin D status. At present no study has investigated elite athletes’ attitudes to sun exposure and skin cancer. Sun safety messages are commonplace in New Zealand and Australia due to the high rates of skin cancer in these countries from sun exposure [8-10]. Outdoor athletes may have a increased risk of skin cancer as they spend many hours training in the sun [11-13], are limited in the sun protective clothing they are able to wear [13,14], and often cannot seek shade during training or games, with the effectiveness of sunscreen reduced by sweating [13,15] all factors which increase the risk of skin cancer. Therefore these athletes are likely to be at higher risk of skin cancer than the general population. On the contrary, information about Vitamin D is not as strongly promoted and is much less understood by the public [8,9]. It is therefore important to understand an athletes knowledge and concerns for Vitamin D and sun exposure prior to initiating educational programs.

## Methods

110 elite outdoor athletes volunteered and provided written, informed consent to participate. Elite was defined as competing internationally. Ethical approval was obtained from the University of Otago Ethics Committee before athletes were approached to participate in the study. Athletes were recruited from rugby, field hockey and rowing and all provided written consent prior to any data collection. Data was collected at the team training bases located around 37° latitude (Hamiliton, Auckland and Lake Karapioro) during a New Zealand summer.

Participants were interviewed in private on topics including Vitamin D and sun exposure. In total there were 57 multiple-choice or short answer questions based on previous questionnaires [16-19]. The interview sessions were peer reviewed by a registered sports dietitian and pilot tested for clarity via a focus group prior to the main study.

Participants were asked to identify their ethnicity (self-selected based on the New Zealand census) [20]. Athletes were also asked to describe the colour of their natural, untanned skin using the Fitzgerald scale [21].

Knowledge of vitamin D was initiated by first asking the participants if they “had ever heard of vitamin D?”, followed by questions “are you able to name any health benefits of vitamin D?”, and “do you know any personal characteristics that could affect an individual’s vitamin D levels?”. A knowledge score was created by summing the number of correct responses for both these questions. Participants were not informed if their responses were correct and no help or prompts were provided by the interviewer.

Participants were also asked “are you concerned about your Vitamin D status?”

A section of the questionnaire was designed to assess athletes attitudes towards sun exposure. This section included questions on the participant’s concern about their risk of skin cancer from sun exposure, concern about their vitamin D status and whether they intentionally spend time in the sun to improve their vitamin D status or to tan; sunscreen use and, if appropriate, reasons for not using sunscreen or the sunscreen protection factor and reapplication were asked; clothing during training sessions including questions on sunglasses and hat use was collected.

Statistical analyses were undertaken using Stata 12.0 (StataCorp, USA). Chi-squared tests were used to determine if answers to questions on knowledge of vitamin D, attitudes to vitamin D and sun exposure differed between sports, ethnicity and gender. Where significant differences (p < 0.05) within groups were found, post-hoc binomial probability tests were used to determine whether there were proportional differences in the answers given between each of the three sports and four ethnic groups. Bonferroni adjustment was used to account for multiple comparisons. The Kruskal-Wallis test was used to determine differences between sport, ethnicity and gender for the questions related to sun exposure and sunscreen use which had five possible answers “Never”, “Rarely”, “Sometime”, “Often”, “Always”. The data are presented as the number of participants (percentage of that category of participants) unless otherwise stated.

## Results

The characteristics of athletes are shown in Table 1. Ethnic groups were; New Zealand (NZ) European, Maori, Pacific Island and “Other” which included all other ethnicities.

**Table 1:** **Number (%) of participants in each gender, ethnicity, skin colour and sport category and mean (±SD) age (years)**

**Characteristic**	**Category**	**All** ***n*** **= 110**
Gender*	Male	76 (69%)
	Female	34 (31%)
Age (years)^†^		23.53 ± 3.11
Ethnicity*	NZ European	76 (69%)
	Māori	19 (17%)
	Pacific Island	11 (10%)
	Other	4 (4%)
Skin Colour*	Fair	35 (32%)
	Medium	42 (38%)
	Olive	18 (16%)
	Dark	15 (14%)
Sport*	Rugby	35 (32%)
	Hockey	22 (20%)
	Rowing	53 (48%)

*number (%), † mean ± SD.

The majority 107 of the 110 (97%) athletes reported that they had heard of vitamin D and there were no significant differences between these groups.

84 (76%) of athletes were able to identify the sun as source of vitamin D as shown in Table 2. There were differences in ethnicity (p < 0.001) as more NZ European and Māori athletes knew the sun was a source of vitamin D compared to Pacific Island athletes (p < 0.008). However, only 18 (17%) of the total sample was able to name another source of vitamin D such as food sources (6%) or supplements (10%). Examples of the correct food sources the participants identified included: milk, meat, fish and eggs. Incorrect answers included a variety of fruits and vegetables. One athlete correctly mentioned sunbeds as a source. There were no differences between males and females or the four ethnicities in athletes’ knowledge of sources of vitamin D, other than the sun. However, there was a difference between sports (p = 0.038) with rowers more likely to know another source than hockey players (p = 0.017).

**Table 2:** **Proportion of athletes able to name a source of Vitamin D**

	**Able to name the sun as a source of vitamin D**		**Able to name another source of vitamin D**	
	**n (%)**	**p-value^**	**n (%)**	**p-value^**
All	84 (76%)		19 (17%)	
Gender		0.323		0.634
Male	56 (74%)		14 (18%)	
Female	28 (82%)		5 (15%)	
Ethnicity		<0.001		0.119
NZ European	64 (84%)		13 (17%)	
Māori	16 (84%)		6 (31%)	
Pacific Island	2 (18%)*^1,2^		0	
Other	2 (50%)		0	
Sport		0.058		0.038
Rugby	22 (63%)		6 (17%)	
Hockey	17 (77%)		0	
Rowing	45 (85%)		13 (25%)*^3^	

n = 110.

*p < 0.05 with Bonferroni adjustment.

^Chi-squared test.

^1^Significant difference between NZ European and Pacific Island ethnicity (p < 0.05).

^2^Significant difference between Māori and Pacific Island ethnicity (p < 0.05).

^3^Significant difference between Rowing and Hockey (p < 0.05).

Almost half (n = 45) (45%) of the sample who had heard of vitamin D were able to correctly name at least one personal characteristic that affects vitamin D status such as: skin colour (n = 30, 27%), sun exposure (n = 22, 20%), diet (n = 9, 8%), body fat (n = 2, 2%) and age (n = 2, 2%). Fourteen percent of athletes could name two or more characteristics and although three quarters of these athletes were rowers this was not a significantly different between sports. There were no differences in athletes’ knowledge of personal characteristics affecting vitamin D status according to gender, sport or ethnicity.

Of those athletes who had heard of vitamin D, one quarter (n = 27, 25%) were able to name at least one health benefit of having an adequate vitamin D status. The health benefits named included: bone health (n = 18, 16%), immunity (n = 7, 6% of athletes), mood (n = 6, 5%), and muscle strength (n = 2, 2%).

Many of those who named bone health as a benefit were rowers (94%). There was a significant difference in knowledge of the health benefits of having an adequate vitamin D status between sports (p = 0.031) with rowing having greater knowledge than hockey.

One third of athletes (n = 36, 33%) reported intentionally spending time in the sun in order to get a tan and two thirds (n = 74, 66%) were concerned about the risk of skin cancer when exposing their skin to the sun as shown in Table 3. This compared to only 6% (7 athletes) who were concerned with their Vitamin D status. There were gender differences (p < 0.01), sport differences (p < 0.01) and ethnic differences (p = 0.031) for the athletes that spent time in the sun to tan. Over half of female athletes (n = 22) spend time in the sun to tan in comparison to 18% (n = 14) of males (p < 0.01). No Pacific Island athletes reported spending time in the sun to tan compared to 65% (n = 31) of NZ Europeans (p < 0.01). Hockey players (n = 16, 73%) were significantly more likely to spend time in the sun to tan than rowers (n = 14, 26%) or rugby players (n = 6, 17%) (p < 0.01). Male hockey players reported spending more time in the sun than male rowers and rugby players (p < 0.001). In addition, female hockey players spent more time in sun to tan than female rowers (p = 0.018).

**Table 3:** **Elite athletes’ attitudes towards sun exposure and differences between gender, ethnicity and sport n = 110**

	**Spend time in the sun to tan**		**Concerned about risk of skin cancer with sun exposure**	
	**Yes n(%)**	**p-value^**	**Yes n(%)**	**p-value^**
Total	36 (33%)		73 (66%)	
Gender		<0.01		0.806
Male	14 (18%)		51 (67%)	
Female	22 (65%)		22 (65%)	
Ethnicity		0.031		0.430
NZ European	31 (41%)^1^		53 (70%)	
Māori	4 (21%)		12 (63%)	
Pacific Island	0		5 (45%)	
Other	1 (25%)		3 (75%)	
Sport		<0.01		0.047
Rugby	6 (17%)		18 (51%)^4,5^	
Hockey	16 (73%)^2,3^		18 (82%)	
Rowing	14 (26%)		37 (70%)	

^Chi-squared test.

^1^Significant difference between NZ European and Pacific Island ethnicity (p < 0.05).

^2^Significant difference between Hockey and Rugby (p < 0.05).

^3^Significant difference between Hockey and Rowing (p < 0.05).

^4^Significant difference between Rugby and Hockey (p < 0.05).

^5^Significant difference between Rugby and Rowing (p < 0.05).

As displayed in Table 3, there was a difference between sports in those that were concerned about their risk of skin cancer with sun exposure (p = 0.047). Half (n = 18) of rugby players were concerned about the risk of skin cancer when exposed to the sun which was significantly less than the proportion of concerned hockey players (n = 18, 82%) or rowers (n = 37, 70%) (p < 0.01). There was no difference between gender or ethnicity.

Ninety-nine of 110 athletes reported using some sunscreen, however only 10 (9%) athletes reported that they always apply sunscreen before going out in the sun. There was a significant difference between ethnicity for sunscreen use with those of Pacific Island ethnicity reporting less sunscreen use compared to the NZ European (p < 0.001) and tending to less regularly use sunscreen compared to NZ Maori (P = 0.076). No participant reported reapplying sunscreen every hour, 5 reported reapplying it at breaks in training and the same number only if they were reminded to do so. A further 8 (7%) reported reapplying sunscreen if they felt their skin burning and another 15 saying they only did it randomly when they remembered. However, 48 (44%) reported that they never reapply sunscreen and 25 (23%) did not know how regularly they reapplyed sunscreen. Of those that were concerned about skin cancer (n = 73), only 45% (n = 33) reported using sunscreen always or most of the time, which was not significantly different (p = 0.312) to those that were not concerned about skin cancer (n = 37), with 13 athletes (35%) reporting that they used sunscreen most or all of the time. There was no difference in sunscreen use by gender (p = 0.094) nor by sport when controlling for ethnicity (p = 0.471). The majority of athletes stated that they used an SPF30 for protection (range SPF15-100). Time and availability were the two main reasons provided for not applying sunscreen with 75 (68%) and 67 (61%) athletes respectively providing these as a barrier to sunscreen use. Despite lack of availability being cited as a reason for not using sunscreen 65 athletes did state that it was available at training. Sunglasses were always worn by 40 athletes but 36 responded that they never wear sunglasses. Only two athletes stated that they always seek the shade and 68 never actively seeking the shade for protection. NZ European sought shade more frequently than Maori (p = 0.028) and Pacific Island (p = 0.001). There was also a gender difference with females less frequently seeking shade than males (p < 0.001). In terms of sun protection for the head only one athlete always wore a hat and 56 never wore one. The most common type of hat was a baseball type cap.

## Discussion

This study showed that more elite New Zealand athletes were concerned about their future skin cancer risk than concerned about their vitamin D status. Yet despite their skin cancer concerns, the athletes’ practices in the sun, especially around sunscreen re-application could be elevating their risk of skin cancer.

It seems that they are more aware of Vitamin D than the general population. Almost all (97%) athletes in this study had heard of vitamin D, in comparison, published literature from the general population reports only 69-84% have heard of vitamin D [16,19,22]. The differences between the general population and this athletic population may reflect the dietetic and medical support available to these elite athletes, which is less available to the general population. Future studies should investigate the source of information. Further, athletes generally had good knowledge of the sun as a source of vitamin D (76%) which was similar to previous findings in the general population [16,22,23]. However in contrast to the published research in the general population, a smaller proportion of participants could name a correct food source or supplements as a source of vitamin D in the present study [16,22,24]. Although vitamin D intake from food sources alone would not provide sufficient vitamin D for optimal serum concentrations, intake from food could assist when sun exposure is limited. Elite New Zealand athletes have greater access to nutritional support and education than the general population and given the potential role for Vitamin D in health and performance it is interesting that their knowledge of food sources of Vitamin D was not higher than the general population. However, these results must be interpreted with caution as the type of questionnaire used to assess knowledge may influence the scores. The present study utilised an open ended question style whereas others have used a multi-choice type questionnaire [16,22], which generally overestimated the participants knowledge. When groups are asked prompted questions they seem to have a greater knowledge of vitamin D [16,19,22] than when asked open-ended questions [19,22], indicating the ability to express vitamin D knowledge might be highly dependent on the question format. This is highlighted by Kung and Lee [19] who asked older (>50 years) Chinese women either open-ended questions or prompted responses about Vitamin D, the proportion of correct responses varied depending on the type of questioning [16,22]. Only one-quarter named the sun as a source of vitamin D, less than 10% named correct food sources and less than 1% were aware vitamin D could be obtained from supplements. Interestingly, when they were then prompted, responses were inconsistent as over half of the group claimed to know the sun is a source of vitamin D. Furthermore, caution must be taken when comparing studies as the results may reflect the sun smart messages individual to each country [9,23]. Regardless of previous studies, athletes’ vitamin D knowledge was poor especially when considering the potential risk of low vitamin D status to health and sporting performance [25].

Despite the role vitamin D plays in health, only a small percentage of athletes were concerned about vitamin D status (6%) which is similar to studies in the general population in which 9% [16] and 12% [22] were concerned with their vitamin D status. In the current study, those who had a greater knowledge score were concerned about vitamin D. Therefore they may have understood their risk of deficiency and educated themselves on potential solutions. Another study [24] found those who knew more about the sources of vitamin D tended to consume more vitamin D-containing foods and supplements. Therefore educating athletes about the importance of vitamin D for helath and importance may increase concern for their vitamin D status and subsequently, behaviours that have the potential to improve vitamin D status, either through food, supplementation, safe sun exposure or a combination of factors, will be undertaken. There have been no studies to date investigating the effectiveness of educational interventions on behaviour change and vitamin D status.

The proportion of athletes who intentionally spend time in the sun to improve their vitamin D status (12%) was similar to the general population (9%) of New Zealand [26]. One Australian study [22] found 16% of participants intended to increase their sun exposure due to concerns about their vitamin D status and 21% had already changed their behaviour. This practice could increase the risk for skin cancer; which is well-known to be associated with sun exposure [27]. Advice to athletes regarding Vitamin D should be provided alongside messages about sunscreen use to ensure athletes are not further increasing their risk of skin cancer in attempt to improve vitamin D status [9,28].

In contrast to the number of athletes concerned with their vitamin D status, a greater number were concerned about skin cancer as the result of sun exposure (66%), this possibly reflects the strongly promoted sun safety messages and the high prevalence of skin cancer in New Zealand [29,30]. The high rate of concern for skin cancer may be the reason for the high proportion of athletes who report sunscreen use. This suggests an awareness of sunscreen use to attenuate the risk of skin cancer. However, the lower proportion who use sunscreen all the time and the even smaller amount who regularly reapply sunscreen is of concern especially given the higher risk of skin cancer in New Zealand [29,30]. The data suggest that even those who are concerned about skin cancer risk are not fully aware of the optimal application of sunscreen. In order to promote a more effective use of sunscreen ie frequent reapplication, the reasons for not using sunscreen need to be addressed. Factors such as ensuring sunscreen is available at the training location and that the athletes are provided with training breaks in order to reapply sunscreen along with towels to wipe excess cream off their hands therefore minimizing the impact on training could help promote sunscreen use and attenuate the risk of skin cancer. Factors such as seeking shade and the wearing of sunglasses maybe more difficult to address in some sports due to the training venue or sporting regulations, however, in such cases education about attenuating the risk of skin cancer whilst away from training may be beneficial.

## Conclusion

The current guidelines in New Zealand and the United Kingdom suggest casual summer sun exposure is enough to maintain vitamin D in a healthy population [9,12,28], therefore there is no reason that sun safety messages and vitamin D cannot be promoted in unison.

## Abbreviations

UVB: UltraViolet B
NZ: New Zealand
SPF: Sun protection factor
